# A Sandwich-Structured Piezoresistive Sensor with Electrospun Nanofiber Mats as Supporting, Sensing, and Packaging Layers

**DOI:** 10.3390/polym10060575

**Published:** 2018-05-23

**Authors:** Zicong Zhao, Bintian Li, Liqun Xu, Yan Qiao, Feng Wang, Qingyou Xia, Zhisong Lu

**Affiliations:** 1 Chongqing Key Laboratory for Advanced Materials & Technologies of Clean Energies, Southwest University, 1 Tiansheng Road, Chongqing 400715, China; zhaozc1993@163.com (Z.Z.); libint0506@163.com (B.L.); xulq@swu.edu.cn (L.X.); Yanqiao@swu.edu.cn (Y.Q.); 2 Institute for Clean Energy & Advanced Materials, Faculty of Materials & Energy, Southwest University, 1 Tiansheng Road, Chongqing 400715, China; 3 State Key Laboratory of Silkworm Genome Biology, Southwest University, Chongqing 400715, China; wangf1986@swu.edu.cn (F.W.); xiaqy@swu.edu.cn (Q.X.); 4 Chongqing Engineering and Technology Research Center for Novel Silk Materials, Southwest University, Chongqing 400715, China

**Keywords:** piezoresistive sensor, electrospinning, wearable electronics, human motion, vital sign

## Abstract

Electrospun nanofiber mats have been used as sensing elements to construct piezoresistive devices due to their large surface area and high porosity. However, they have not been utilized as skin-contact supporting layers to package conductive nanofiber networks for the fabrication of piezoresistive sensors. In this work, we developed a sandwich-structured pressure sensor, which can sensitively monitor human motions and vital signs, with electrospun nanofiber mats as supporting, sensing, and packaging layers. The nanofiber mats were prepared by electrospinning with biocompatible poly (l-lactide) (PLA), silk fibroin (SF), and collagen (COL) as raw materials. The synthesized PLA–SF–COL mat possesses a non-woven structure with a fiber diameter of 122 ± 28 nm and a film thickness of 37 ± 5.3 μm. Polypyrrole (PPy) nanoparticles were grown in-situ on the mat to form a conductive layer. After stacking the pristine and conductive mats to form a PLA–SF–COL mat/(PPy-coated mat)_2_ structure, another layer was electrospun to pack the multilayers for the construction of a sandwich-structured piezoresistive sensor. The as-prepared device can sensitively detect external pressures caused by coin loading and finger tapping/pressing. It can also tolerate more than 600 times of pressing without affecting its sensing capability. The human body-attached experiments further demonstrate that the sensor could real-time monitor finger/arm bending, arterial pulse, respiration rate, and speaking-caused throat vibration. The electrospinning-based fabrication may be used as a facile and low-cost strategy to produce flexible piezoresistive sensors with excellent skin-compatibility and great pressure sensing capability.

## 1. Introduction

A wearable electronic device has been regarded as the best choice for everyday health monitoring due to its superior capability to real-time detect vital signs such as pulse rate, respiration rate, body temperature, and blood pressure [1,2,3,4,5,6,7,8,9]. The device can efficiently convert human physiological parameters into electronic signals, which are detectable, recordable, and analyzable with miniaturized instruments. Generally, wearable electronic pressure sensors can be divided into three types according to their working mechanisms: capacitive, piezoelectric, and piezoresistive sensors [10,11,12,13,14]. A microfabrication technique is needed to precisely control the opposite area and distance between the two electrodes in capacitive pressure sensors [10]. Although these sensors may provide relative high sensitivity, the complicated fabrication process greatly hinders their large-scale production and practical applications. Piezoelectric materials such as ZnO nanorod arrays and polyvinylidene fluoride (PVDF) films are used to functionalize flexible substrates for the preparation of piezoelectric sensors [5,11,12,13,14,15], which can generate electric charges in response to the applied mechanical stress. However, the construction of highly oriented 1-dimensional nanostructures and the adjustment of the polymer crystalline phase are very critical for the achievement of good piezoelectric effects.

In the past few years, piezoresistive pressure sensors based on flexible supports have been investigated as attractive wearable devices for human vital signs monitoring [16,17,18,19,20]. A typical one is fabricated by stacking flexible substrates and conductive sensing layers to form a sandwiched structure. Various materials including metal nanoparticles [16], carbonized fabrics [17], carbon nanofibers [18], and polymers [19] have been utilized to prepare the conductive sensing layers in the sandwich-structured sensors. Under mechanical stress, the conductive layers are compressed to create more crosslinking points for fast electron transfer, resulting in device resistance reduction. Since micro- and nano-scale fibers could generate a large number of potential networking sites, conductive fiber networks were produced to enhance the sensing performance [14,21,22].

The electrospinning technique is a facile and low-cost method to prepare nanoscale non-woven fiber scaffolds with high surface area and large porosity [23,24,25,26,27,28,29,30,31,32,33]. It has also been applied to construct piezoresistive sensors containing fiber network layers. Electrospun nanofibers have been assembled to produce 3-dimensional and ultralight sponges with tunable conductivity for a tactile pressure sensor [34]. Poly(3,4-ethylenedioxythiophene):poly(styrenesulfonate)–polyvinyl alcohol nanofibers have been electrospun on a Kapton substrate and encapsulated with polydimethylsiloxane (PDMS) to construct a piezoresistive strain sensor. The device could be stuck on a human finger for the detection of finger bending [21]. Electrospun poly(4-vinylpyridine) nanofiber mats have been coated with a polyethylene terephthalate (PET) film and further micropatterned by lithography for the development of piezoresistive strain sensors [35]. A highly sensitive piezoresistive sensor consisting of PET substrate, electrospun polyaniline nanofibers, and Au-coated PDMS micropillars has been fabricated to measure neck pulse and wrist pulse [36]. Very recently, a freestanding carbon nanofiber mat, which was synthesized by carbonizing electrospun polyacrylonitrile network, was embedded with two layers of polyurethane to preparea piezoresistive sensor for human motion monitoring [18]. Unfortunately, besides electrospun nanofiber mats, flexible polymer substrates and packaging layers have been employed in all those reported devices.

To effectively detect human vital signs, the piezoresistive sensor has to be attached on human skin to sense the extremely weak vibration caused by blood flow and respiration. Therefore, in addition to the pressure sensing performance, skin affinity should be considered in device design. Polymers such as PET and PDMS could provide excellent flexibility and processability, but their biocompatibility and air/moisture permeability are still questionable. Electrospun nanofiber mats consisting of biocompatible polymers and natural proteins have already been utilized as degradable scaffolds for cell adhesion and proliferation in tissue engineering [37]. Moreover, their porous structures may enable the exchange of air/moisture between the skin and the environment. Electrospun nanofiber mats should be ideal supporting layers for skin-attached applications. However, to the best of our knowledge, electrospun nanofiber mats have not been utilized as skin-contacting layers to sandwich conductive nanofiber networks for a piezoresistive sensor.

In this work, we fabricated a flexible, sensitive, and skin-compatible sandwich-structured piezoresistive pressure sensor with electrospun fiber mats as supporting, sensing, and encapsulating layers. The substrate and packaging layers were electrospun nanofiber films consisting of eco-friendly poly(l-lactide) (PLA), collagen (COL), and silk fibroin (SF). The active layers are PLA–SF–COL films coated with polypyrrole (PPy). The surface morphologies and components of the films were characterized with scanning electron microscopy (SEM), X-ray diffraction (XRD), and attenuated total reflection–Fourier transfer infrared spectroscopy (ATR–FTIR), respectively. The pressure sensing property and durability were tested to estimate the performance of the as-prepared devices. The real-time measurements of human motions (finger/arm bending), vital signs (wrist/neck pulse and respiration rate), and throat vibration caused by speaking were conducted to demonstrate the great potential of the electrospun nanofiber mat-based pressure sensors in practical applications.

## 2. Materials and Methods

### 2.1. Chemicals

PLA pellets (*M*w = 150 kDa, 99%), SF (*M*w = 50 kDa, 99.99%) and COL (*M*w = 120 kDa, 99%) were kindly provided by the Department of Orthopedics, Chongqing Hospital of Traditional Chinese Medicine. Pyrrole (Py, 99%) was purchased from Adamas-beta (Shanghai, China). Iron chloride hexahydrate (FeCl_3_∙6H_2_O, 99%), *p*-toluenesulfonic acid sodium salt (96%) and 1,1,1,3,3,3-hexafluoro-2-propanol (HFIP, 99.5%) were bought from Aladdin Reagent Co. (Shanghai, China). All other chemicals were of analytical grade and directly used without further purification in this study. Deionized water was produced by a Millipore water purification system.

### 2.2. Preparation of PLA–SF–COL Nanofiber Mats by Electrospinning

**Scheme 1:**
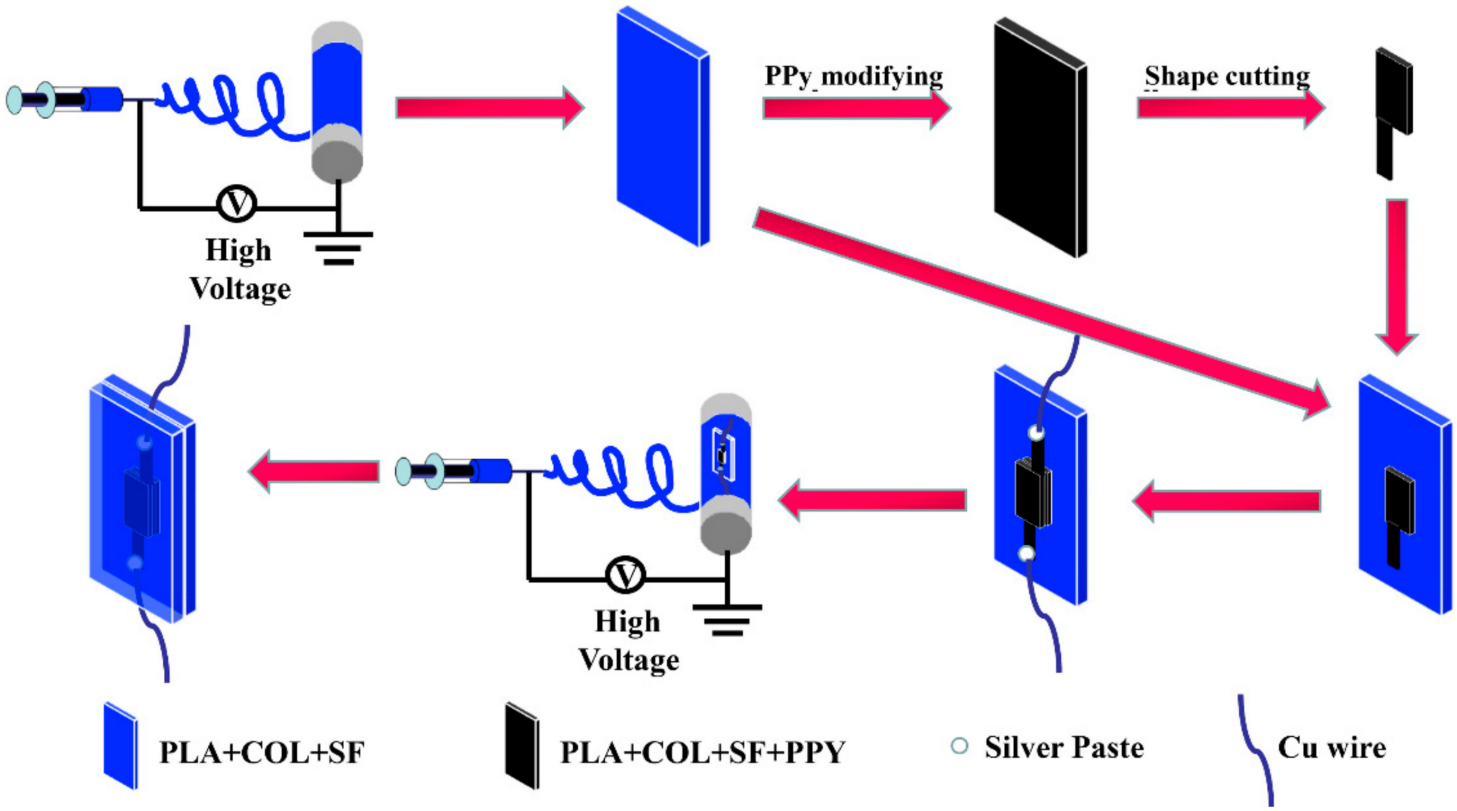
Fabrication of a piezoresistive sensor based on electrospun nanofiber mats.

The PLA–SF–COL nanofiber mats were prepared by the Wang’s route [37]. PLA pellets, SF, and COL were successively added into HFIP at weight percentages of 4%, 2%, and 2%, respectively [37]. The mixture was stirred at room temperature for 6 h to thoroughly dissolve the above solutes. A microinjection pump, a high-voltage power supply, and a grounded drum were used to construct a homemade electrospinning system. The as-prepared precursor solution was fed into a blunt needle with an inner diameter of 0.6 mm at a constant speed of 0.5 mL/h under a 20 kV voltage. The grounded drum with a diameter of 8 cm, which rotates at a speed of 1000 rpm/min, was placed 20 cm far from the blunt needle tip to collect the nanofibers (Scheme 1).

### 2.3. Fabrication of the Conductive Layers

The freshly prepared PLA–SF–COL nanofiber mats were removed from the drum and soaked in an aqueous solution containing *p*-toluenesulfonic acid sodium salt (dopant) and iron chloride hexahydrate (catalyst) at room temperature for 2 h. Then, Py monomer was added into the above solution, holding at room temperature for different durations (2 to 10 h) [38]. To optimize the Py amount for the achievement of highly conductive films, the Py concentration in the reaction system was changed from 7.5 to 30 mmol/L, while maintaining the molar proportion of Py, catalyst and dopant as 1:0.91:0.39. The treated mats were washed with deionized water three times to thoroughly remove superfluous polymers. After drying at 40 °C, they were cut into flag-shaped pieces with active area of 1 cm × 1 cm (Scheme 1).

### 2.4. Construction of Sandwich-Structured Piezoresistive Sensors

The designed piezoresistive sensor consists of two supporting films and two conductive layers, all of which are electrospun nanofiber mats. The as-prepared PLA–SF–COL electrospun mat was knifed into a 3 cm × 4 cm rectangle film and fixed on the grounded drum to serve as the first supporting layer. Two pieces of flag-shaped PPy-coated mats were stacked face-to-face to form a sensing element with a size of 1 cm × 1 cm. Copper wires were adhered to the ends of the flags with silver paste for later connecting with the measurement instruments. The sensing element was attached in the center of the first supporting layer. Another layer of PLA–SF–COL mat was spun on top of the sensing elements serving as both second supporting layer and sealing material. After clipping, a final piezoresistive sensor with a size of 3 cm × 4 cm was produced (Scheme 1).

### 2.5. Characterizations and Measurements

Morphologies of PLA–SF–COL nanofiber mats before/after each modification were characterized using a JSM-7600 FESEM (JEOL, Tokyo, Japan). The ATR-FTIR (Nicolet 6700 FTIR, Thermo Electronic Corporation, Waltham, MA, USA) and XRD spectra (XRD-7000, Shimadzu, Japan) were examined to confirm the formation of the nanofiber mats. The sensing performances of the device were collected using an electrochemical workstation (CHI 660E, CH Instrument Company, Shanghai, China). During the measurements, the devices were fixed on the apparatus, which consists of a positioning system and a force sensor (Figure S1). Pressure-induced resistance changes were collected under an operating voltage of 1.0 V to evaluate the sensing performance. Body motions, vital signs, and voices of a female volunteer were real-time measured to demonstrate the feasibility of the sensor. Since the operation voltage was quite low, the current response of the device was set at a level of dozens of microamperes, which would not cause adverse effects to the volunteer.

## 3. Results and Discussion

**Figure 1:**
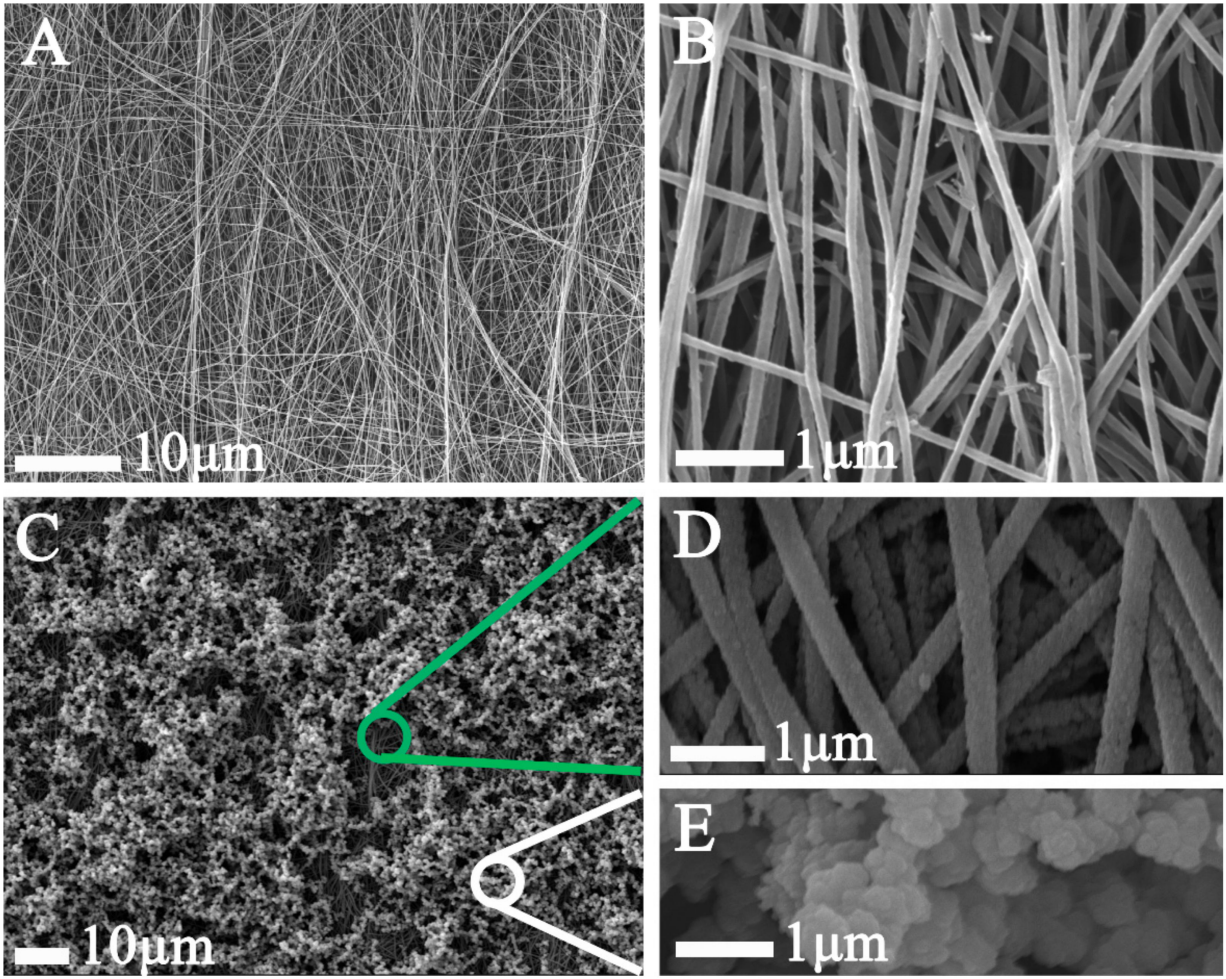
Scanning electron microscopy (SEM) images of an electrospun PLA–SF–COL nanofiber mat at low (**A**) and high magnifications (**B**); (**C**) A PLA–SF–COL nanofiber mat after PPy modification; (**D**–**E**) SEM images of PPy-immobilized PLA–SF–COL nanofiber mat at high magnifications.

PLA, SF, and COL were mixed together to prepare a precursor solution for the electrospinning of biocompatible nanofiber mats. As shown in Figure 1A,B, fibers with an average diameter of 122 ± 28 nm crosslink with each other to form a non-woven structure, which is very typical morphology of an electrospun mat. The thickness of the collected mat was about 37.0 ± 5.3 μm (Figure S2), indicating that the mat could be utilized as a freestanding film for the following PPy modification and sensor assembly. To prepare the sensing layer, the as-prepared mats were immersed in a solution containing catalysts, dopants, and Py monomers for the in-situ growth of PPy. In order to achieve high conductivity, the concentration of Py monomers and the polymerization time were optimized, respectively. It was found that the mats with the highest conductivity could be obtained in the system containing 15 mM Py monomer after 6 h of reaction (Figure S3). The modified mat was covered by agglomerated nanoparticles and the crosslinking fibers could only be seen from the gaps (Figure 1C). Figure 1D,E illustrate the morphologies of PPy-modified nanofibers and PPy stacking on the mats, respectively. In comparison to the relatively smooth surface of PLA–SF–COL fibers, a number of nanoparticles were uniformly coated on the modified fibers (Figure 1D), causing significant increment of the fiber diameter. The amorphous stacking structure could also be observed on the mat surface, which matches well with the morphology of PPy film (Figure 1E) [39]. The cross-sectional SEM image of a modified sample (Figure S4) further confirms the deposition of PPy throughout the whole mat. During the polymerization, PPy may first grow on the fiber surface to form a conductive layer, followed by stacking on the mat to produce amorphous agglomerates (Figure S5). The PPy layers on the fibers and surface-attached PPy agglomerates may both contribute to the conductivity of the PLA–SF–COL-based sensing elements.

**Figure 2:**
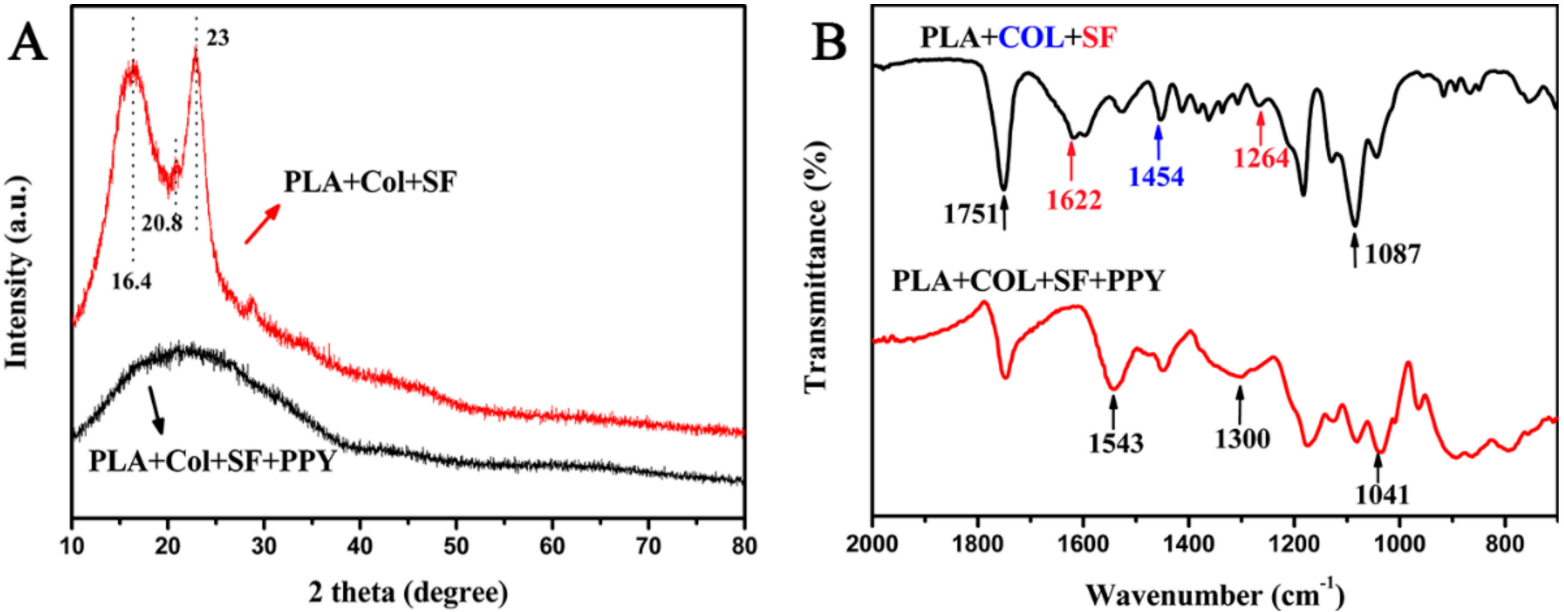
X-ray diffraction (XRD) patterns (**A**) and Fourier transfer infrared spectroscopy (FTIR) spectra (**B**) of PLA–SF–COL nanofiber mats before and after PPy modification, respectively.

XRD and ATR-FTIR were carried out to verify components of the electrospun nanofiber mats and the successful immobilization of PPy. The XRD spectrum of PLA–SF–COL mats (Figure 2A) displays two prominent diffraction peaks at 16.4° and 23°, which can be assigned to the reflection of crystalline PLA and SF, respectively [40,41,42]. No clear peak for COL can be identified from the curve. Normally, COL possesses a wide and weak peak at around 20° [43], which may be masked by the strong diffraction patterns of PLA and SF. As to the PPy-modified mat, a broad peak in the range of 20–30° can be observed, suggesting the coverage of the amorphous polymers on the mat surface [44,45]. Interestingly, the peaks for PLA, SF, and COL could not be found in the XRD pattern of the PPy-modified mats. The phenomenon may be caused by the full coverage of a thick PPy layer on the mats [46,47,48]. Figure 2B shows the FTIR spectra of the electrospun mats before and after PPy functionalization, respectively. The weak bands at 1751 and 1087 cm^−1^ correspond to the backbone ester groups of the PLA [49,50]. The absorptions at 1622 and 1264 cm^−1^ indicate the existence of SF in the mat, while COL contributes to the band at 1454 cm^−1^ [51,52]. After PPy immobilization, some new absorption bands appear in the spectrum. The band at 1543 cm^−1^ could be attributed to pyrrole rings. The peaks at 1300 and 1041 cm^−1^ come from the deforming vibration of N–H and C–H as well as stretching vibration of C–C, respectively [53]. The results undoubtedly prove that the electrospun nanofiber mats are composed of PLA, SF, and COL. After in-situ polymerization, PPy could be deposited on the mats to form fiber-adsorbed and surface-attached structures.

**Figure 3:**
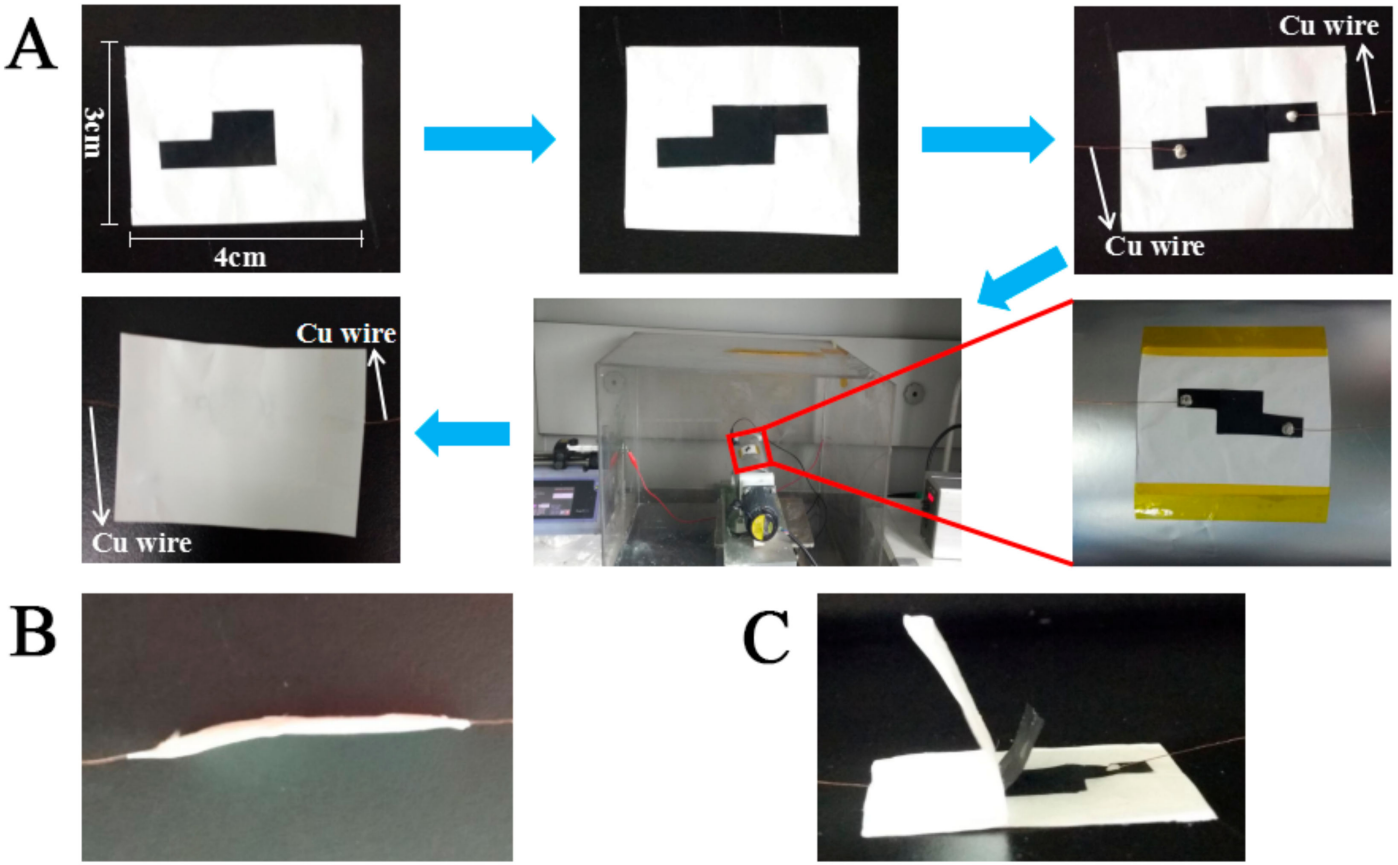
(**A**) Fabrication of a sandwich structured piezoresistive sensor with nanofiber mats as supporting, sensing, and packaging layers via electrospinning; (**B**) Side view of an as-prepared device; (**C**) Side view of a disassembled device.

The as-prepared non-modified and PPy-coated PLA–SF–COL mats were employed to construct sandwich-structured piezoresistive sensors (Figure 3A). The color of the electrospun mats changes from white to black during the PPy immobilization process. After cutting into the desired size and shape, one PPy-functionalized film was attached on a PLA–SF–COL supporting layer. Then, another piece of conductive film was stacked on the top of it to form a sensing element. Two copper wires were adhered at the ends of the top and bottom conductive films, respectively, for later connecting to the testing instruments. The system was fixed on a grounded drum for another round of electrospinning. The deposited nanofiber mat serves as both packaging material and top supporting layer (Figure 3B,C). Finally, a sandwich-structured piezoresistive sensor with nanofiber mats as supporting, sensing, and packaging layers could be obtained for the pressure–response tests and human vital sign detection. The sensing mechanism for the nanofiber mat-based piezoresistive devices could be explained by the change of the number of contacting points during the loading– unloading process (Figure S6). At the unloading state, there are less contact points between the top and bottom conductive layers, thus causing a relatively larger device resistance. On the contrary, external pressure may push the two conductive layers much tighter together to produce more contacting sites, resulting in the reduction of device resistance. 

**Figure 4:**
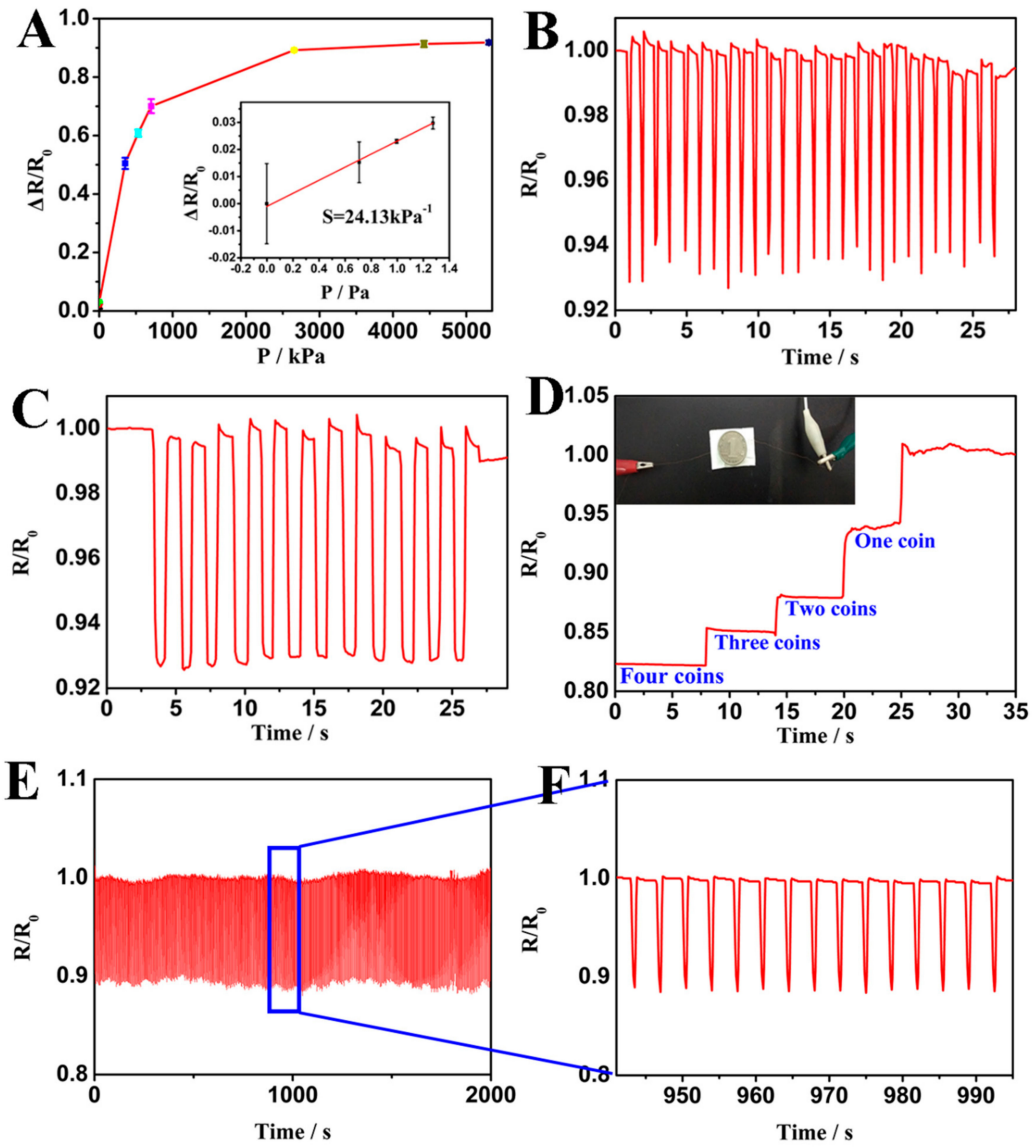
(**A**) Resistance change–pressure relationship; Responses of the sensor to finger tapping (**B**) and pressing (**C**); (**D**) Relative resistance of the sensor after loading different numbers of coins; (**E**) Responses of the nanofiber mats-based piezoresistive sensor upon repetitive loading and unloading of a constant pressure; (**F**) An enlarged view of the selected part.

The pressure-dependent resistance change is exhibited in Figure 4A. In the low-pressure region (from 0 to 1.27 Pa), the resistance change increases linearly with the applied pressure. The sensitivity in this region is as high as 24.13 kPa^−1^ with high linearity of *R*^2^ = 0.99 (Inset of Figure 4A). Based on the experimental data and statistical analysis, the limit of detection was determined to be 1.00 Pa. The current–voltage curves of the device under different pressures show Ohmic behavior (Figure S7). To further investigate the influence of humidity on the device performance, the resistance changes caused by certain pressures under different humidities were tested. As the relative humidity increases from 47% to 56%, the resistance change gradually reduces (Figure S8), suggesting that the quantitative measurement of external pressures should be conducted under an environment with constant humidity. In practical application, the humidity change seems to be inevitable. A re-calibration process may be needed to ensure the accuracy of the quantitative analysis. Human finger tapping and pressing were real-time monitored using the electrospun mats-based sensors with a force at about 3 N (Figure 4B,C). The resistive responses match well with the repetitive tapping and pressing. The external pressure induces a very rapid drop in the device resistance. When the pressure is maintained on the device surface, the resistance stays at a constant level. Once the force is removed, the resistance recovers to the original level. As the tapping frequency increases from 0.1 to 3.5 Hz, the device could provide satisfactory electrical responses (Figure S9). To further demonstrate the sensitivity of the sensor, four coins were stacked on the top of a device and then removed one by one (Figure 4D). The loading of four coins significantly decreased the device relative resistance to ~0.82. During the removal process, the relative resistance gradually increased and finally went back to ~1.0, indicating that the sensor could precisely detect the external pressure change. It was of interest to find that the resistance alterations caused by the 4th and 3rd coins were obviously smaller than those caused by the 2nd and 1st ones. The phenomenon may suggest that the sensor is more sensitive to a relative small pressure. The micro- and nano-scale features of the PPy conductive layers may contribute to the low detection limit, high sensitivity, and fast response of the as-prepared devices. Since durability and stability are critical parameters for a flexible device, responses of the device to repetitive pressing were real-time tested in the present study (Figure 4E,F). The signals perfectly matched with the pressing during the measurement. Moreover, both relative resistances at loading and unloading states kept quite stable at ~0.89 and ~1.0, respectively. The results show that the electrospun mat-based sensors may tolerate more than 600 times of pressing without affecting their pressure sensing capability. To rule out the interference of the piezoelectric effects from PLA, a device without PPy layers was tested. As exhibited in Figure S10, the current signals are negligible in comparison with the ones obtained from a PPy-incorporated device. The sensing performances of three devices were also examined to show the run-to-run difference of the fabrication process (Figure S11). After 7 and 14 days of storage at ambient conditions, there was no significant change on the electric response to a certain pressure, demonstrating the great long-term stability of the as-prepared devices (Figure S12). In our study, we found that both size and shape of the active component could significantly affect the performance of the sensing devices. Further studies may be conducted to systematically investigate the size and shape effects of the active components on the performance of piezoresistive sensors.

**Figure 5:**
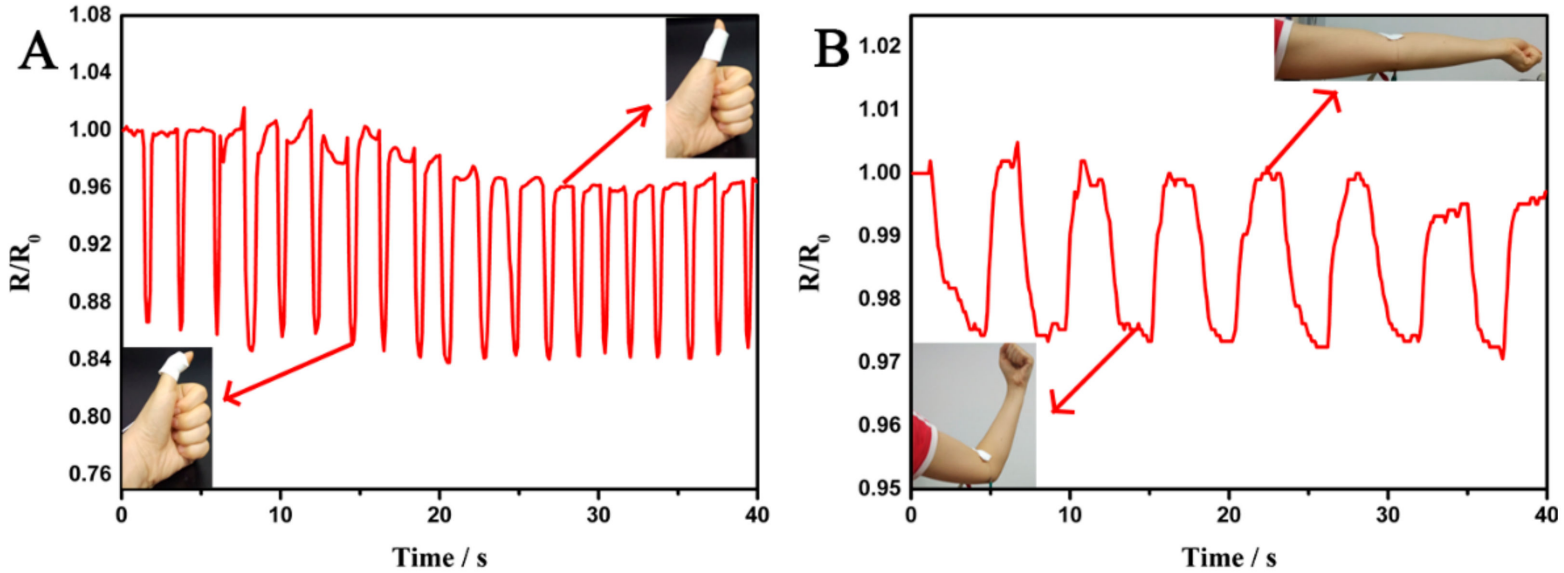
Responses of the nanofiber mats-based piezoresistive sensor to human thumb bending (**A**) and arm bending (**B**).

The electrospun mat-based piezoresistive devices were fastened on the joints of thumb and arm for the measurement of human motions (Figure 5). When the thumb and arm are kept straight, there is no pressure on the attached device and the relative resistance has no alteration. Once the volunteer bends the thumb and arm, extrusion force is applied to the sensor, leading to the sharp resistance decreases observed in real-time curves. As the thumb and arm are straightened again, the device resistances return to the original level. It was noted that the signals may vary in intensity and pattern during the repetitive bending tests, which may have been caused by unstable contact between the two conductive layers.

**Figure 6:**
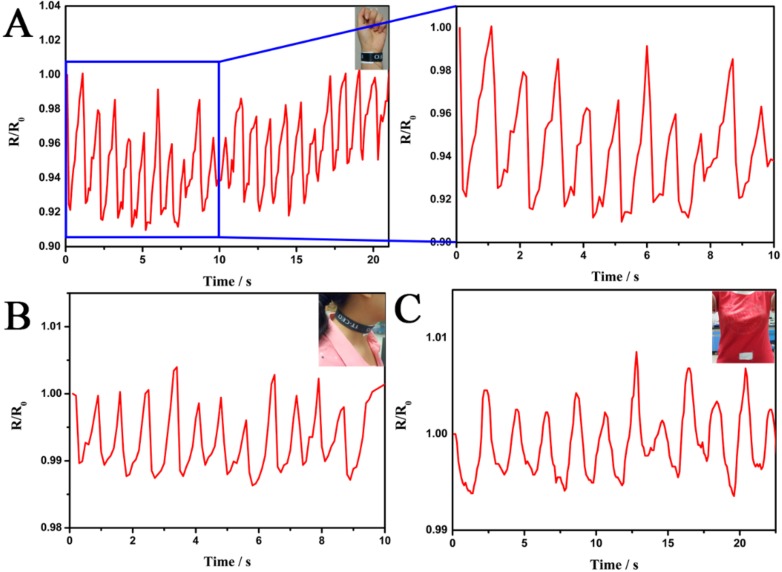
Real-time measurements of human wrist pulse (**A**), neck pulse (**B**) and respiration rate (**C**) using the nanofiber mats-based piezoresistive sensor. Insets: Attachments of the sensor on the volunteer’s wrist, neck, and abdomen, respectively.

Cardiac contraction and relaxation will respectively cause distension and compression of an artery, which may be sensed by the skin-attached sensors. In the present study, the as-prepared devices were mounted on a human wrist and neck for real-time monitoring wrist (Video S1) and neck pulses (Figure 6A,B). The initial resistance of the device was 283.1 ± 0.6 kΩ. The sensor could easily detect signals corresponding to the wrist and neck pulses. Moreover, the wrist and neck pulses could be calculated based on the real-time curves as 66 times/min and 72 times/min, respectively, which fall in the range of a healthy female (60–100 times/min). A control experiment was also conducted in our study for easy comparison (Figure S13). The device was also attached on the volunteer’s abdomen to sense the up and down movement caused by breathing. The resistance reduction and recovery in Figure 6C stand for the inhalation and exhalation of the volunteer, respectively. Within 20 s, 10 occasions of breathing could be detected. The results demonstrate that the electrospun mat-based piezoresistive sensors should be very promising in real-time monitoring of human vital signs such as pulse and respiration.

**Figure 7:**
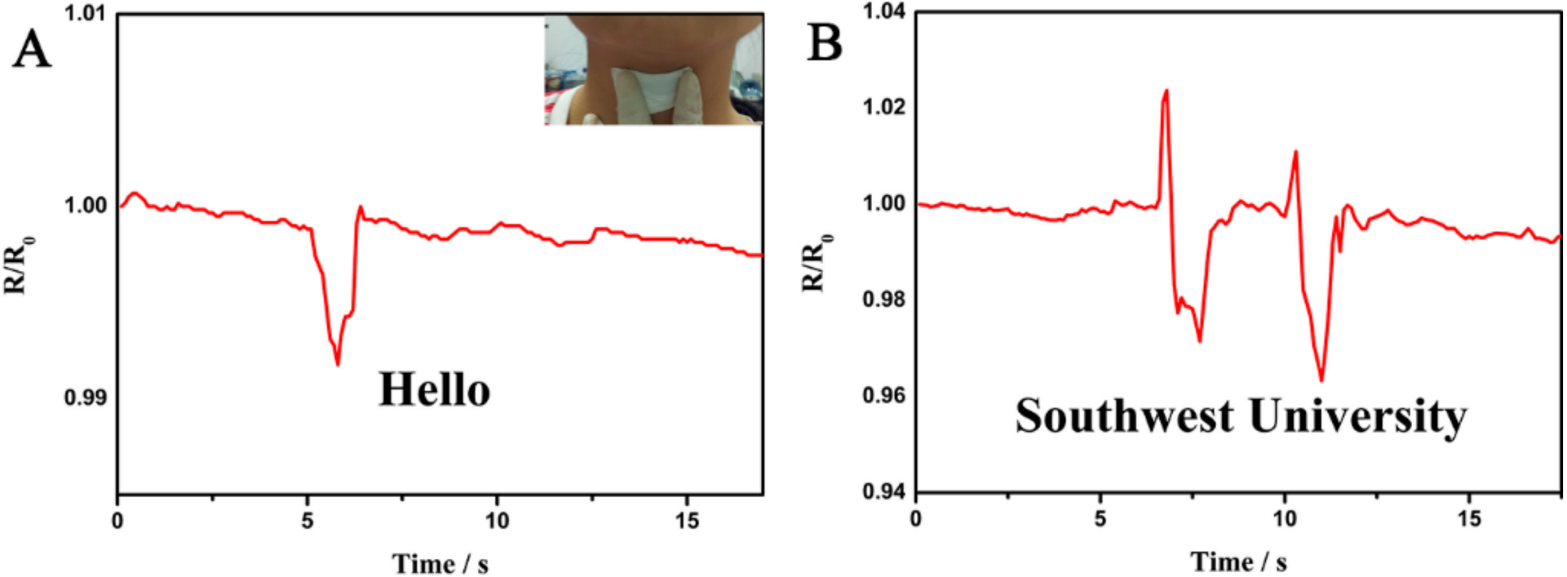
Real-time measurements of vocal-cord vibrations using a throat-attached nanofiber mat-based sensor when the volunteer is speaking “Hello” (**A**) and “Southwest University” (**B**), respectively.

We also tested the responses of the device to the sound change by attaching it on the human throat. When the subject was speaking the word “Hello”, one signal could be immediately discovered in the real-time curve (Figure 7A). However, when the subject was speaking two words “Southwest University”, two well-defined waves, corresponding to the words, appeared (Figure 7B). It is obvious that the sensor could catch the vibration of the throat during the speaking process. Therefore, the sensor may be potentially applied to differentiate human voices and to convert acoustic information into electronic signals in the future.

## 4. Conclusions

In summary, we successfully fabricated a sandwich-structured piezoresistive sensor with electrospun nanofiber mats as supporting, sensing, and packaging layers. Firstly, biocompatible PLA–SF–COL nanofiber mats were prepared via electrospinning to serve as the bottom supporting layers and the matrices for PPy modification. Then, the pre-cut PLA–SF–COL mats were immersed in Py monomer solution for the in-situ growth of conductive polymers. The non-woven structure and the biocompatible components of the mats as well as PPy immobilization were verified with SEM, XRD, and FTIR, respectively. After stacking the materials on the drum to form a PLA–SF–COL mat/(PPy-coated mat)_2_ structure, another PLA–SF–COL mat was deposited on its surface to function as both top supporting layer and packaging layer. The as-prepared device is a freestanding multilayered film with great flexibility and good biocompatibility. After a series of tests, the piezoresistive sensor showed high sensitivity to external pressures and excellent durability to repetitive pressing. With these advantages, it could be applied to monitor finger/arm bending, arterial pulse, respiration rate, as well as speaking-caused throat vibration. This work may not only provide a facile electrospinning-based approach to constructing flexible piezoresistive sensors, but also for use to develop a skin-compatible wearable sensor with great potential in practical real-time monitoring of human motions and vital signs.

## Supplementary Materials

The supporting data are available online at http://www.mdpi.com/2073-4360/10/6/575/s1.
