# Supplementary material for: A Sandwich-Structured Piezoresistive Sensor with Electrospun Nanofiber Mats as Supporting, Sensing, and Packaging Layers
Source: Polymers (Basel). 2018 May 23;10(6):575. doi: 10.3390/polym10060575 (PMC6403861; doi:10.3390/polym10060575)
Supplement: Supplementary file 1 [file polymers-10-00575-s001.zip › Figures S1-13.pdf]

Supporting Information

A Sandwich-structured Piezoresistive Sensor with Electrospun Nanofiber Mats as Supporting, Sensing and Packaging Layers

Zicong Zhao,[ab] Bintian Li,[ab] Liqun Xu,[ab] Yan Qiao,[ab] Feng Wang,[cd] Qingyou Xia[cd] and Zhisong Lu[*ab]

[a.] Chongqing Key Laboratory for Advanced Materials & Technologies of Clean Energies, Southwest University, 1 Tiansheng Road, Chongqing 400715, P. R. China.

[b.] Institute for Clean Energy & Advanced Materials, Faculty of Materials & Energy, Southwest University, 1 Tiansheng Road, Chongqing 400715, P. R. China.

[c.] State Key Laboratory of Silkworm Genome Biology, Southwest University, Chongqing 400715, PR China.

[d.] Chongqing Engineering and Technology Research Center for Novel Silk Materials, Southwest University, Chongqing 400715, PR China.

*: Author to whom correspondence should be addressed. Tel.: +86-23-68254732; Fax: +86-23-68254969. E-mail: zslu@swu.edu.cn (ZS Lu)

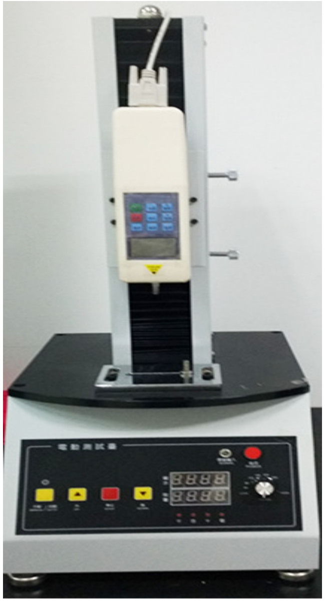

Figure S1. The apparatus used to perform different pressures in this experiment.

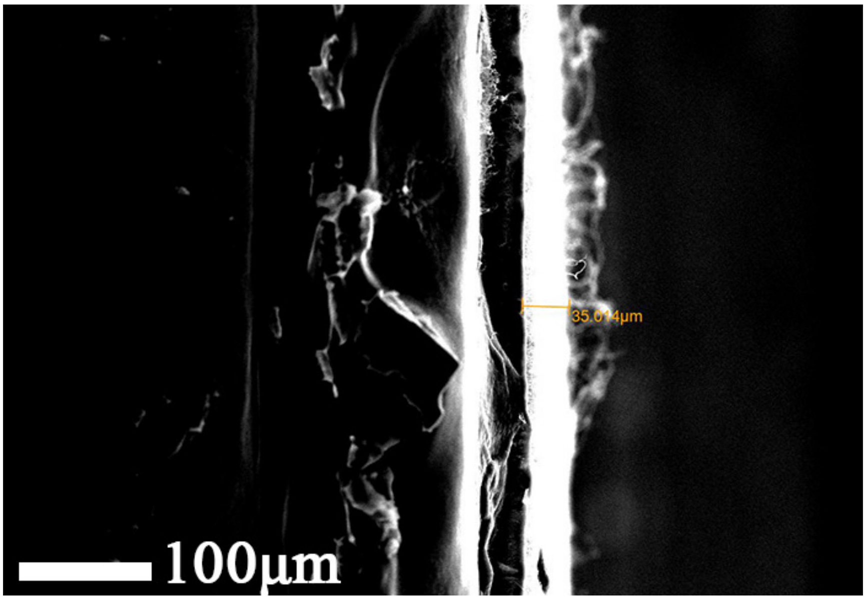

Figure S2. Side-view of a typical PLA-SF-COL nanofiber mat.

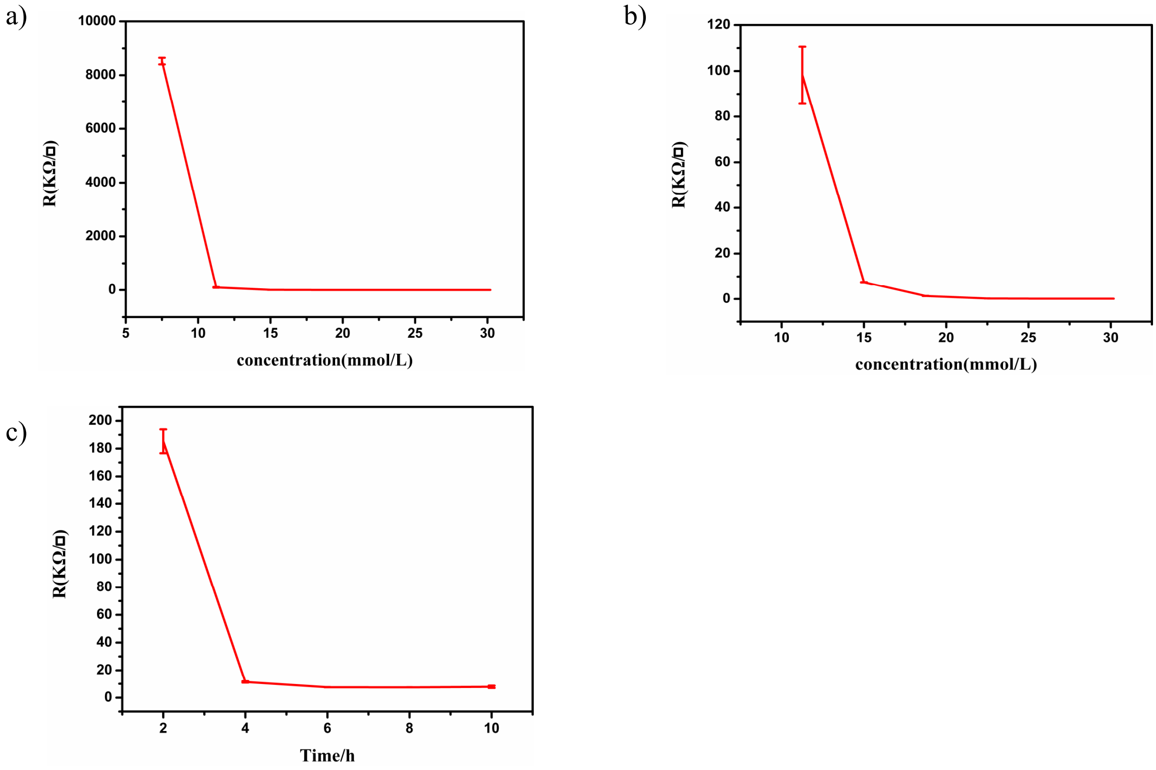

Figure S3. Resistance-concentration curve (a-b) of the conductive layer. (c) Resistance-time curve.

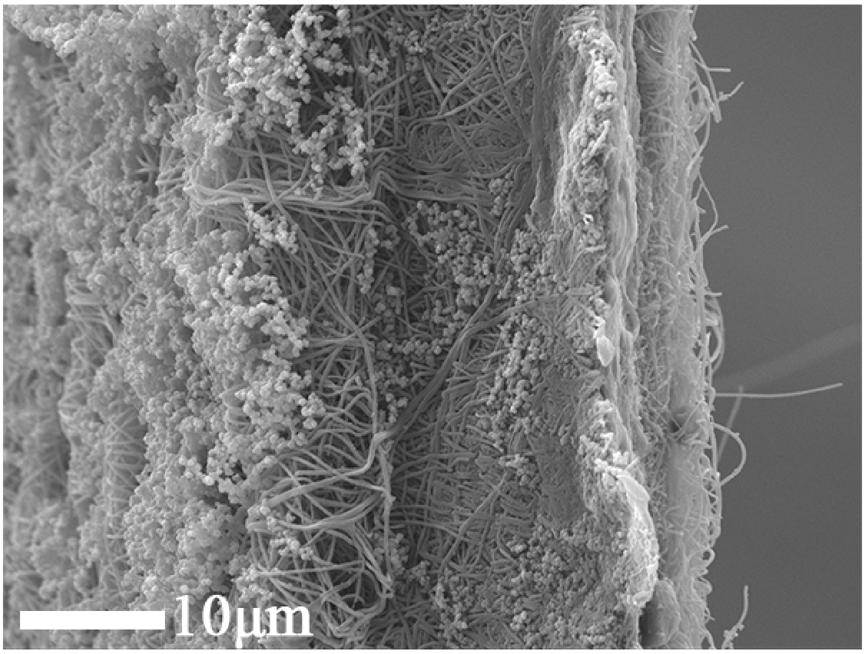

Figure S4. The cross-sectional SEM images of the PPy-modified nanofiber mat.

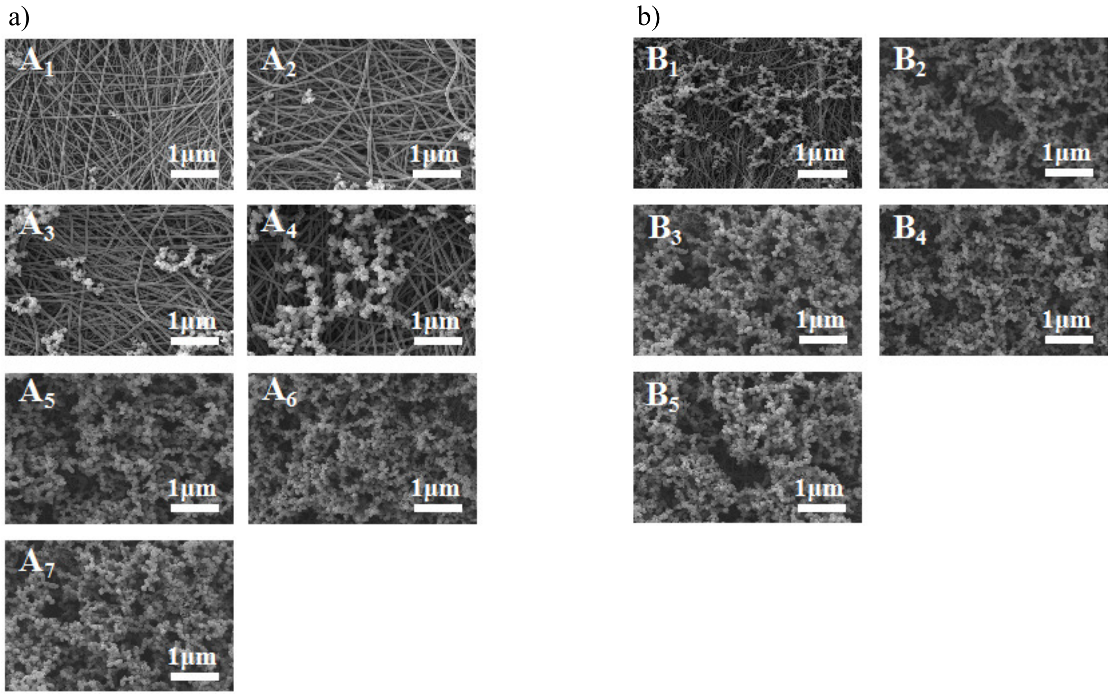

Figure S5. SEM images of (a) optimized Py concentration (A_1-A_7 represent the Py concentration from 7.5 mmol/L to 30 mmol/L, respectively) and (b) different polymerization time (B_1-B_5 represent the polymerization time from 2 h to 10 h, respectively).

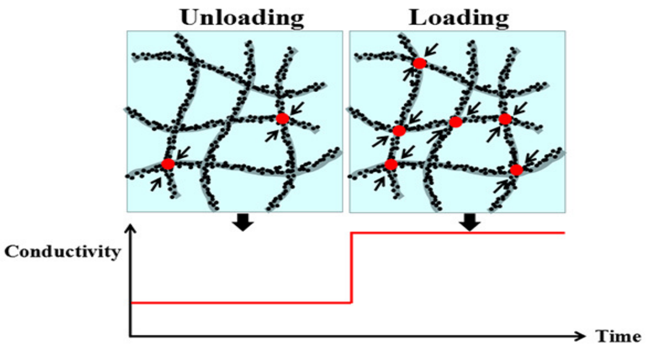

Figure S6. Sensing mechanism for the nanofiber mats-based piezoresistive devices.

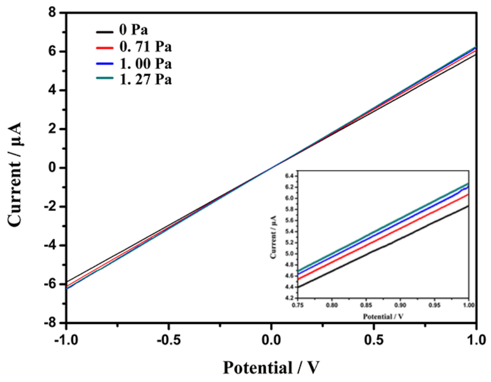

Figure S7. Current-voltage curves of the device under different pressures.

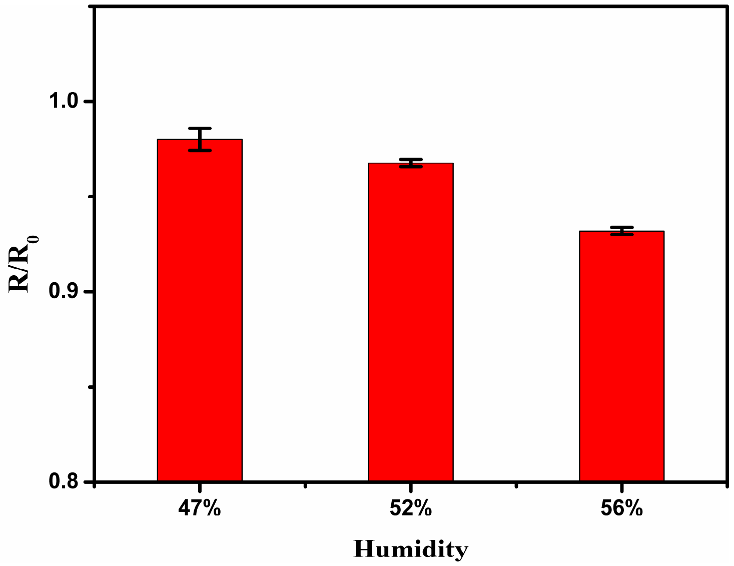

Figure S8. Resistance changes of the device to a certain pressure under different humidity.

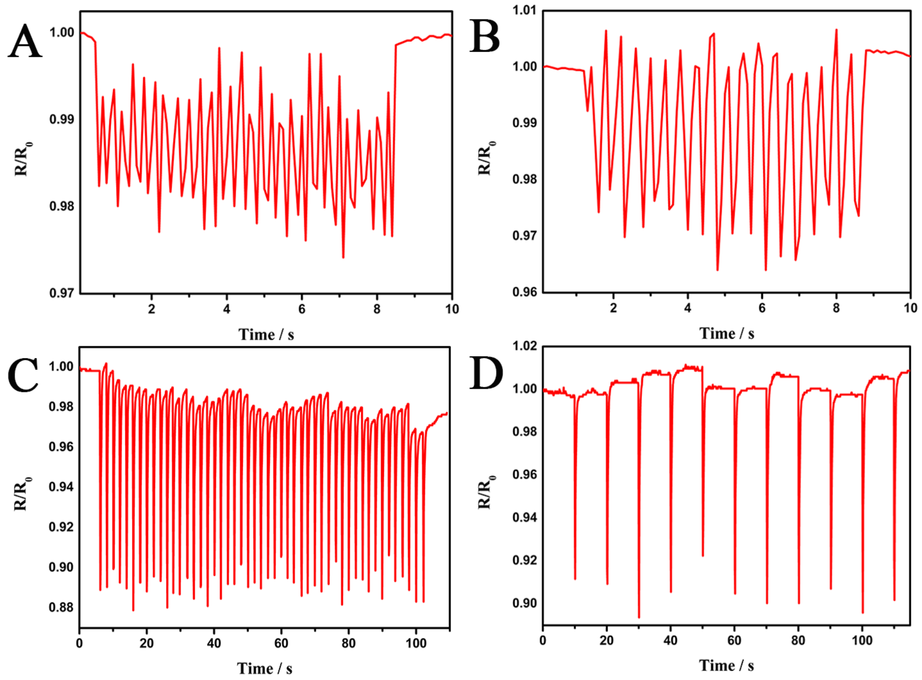

Figure S9. Responses of the electrospun mats-based piezoresistive sensor to tapping with different the frequencies. (A) 3.5 Hz, (B) 2.5 Hz, (C) 0.5 Hz and (D) 0.1 Hz.

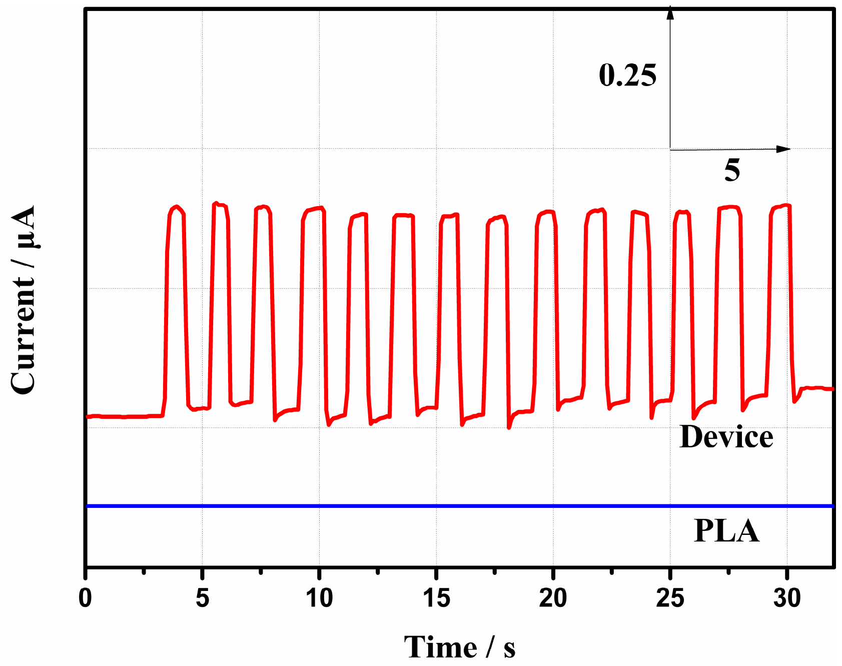

Figure S10. Effect of piezoelectric property of PLA on the device performance.

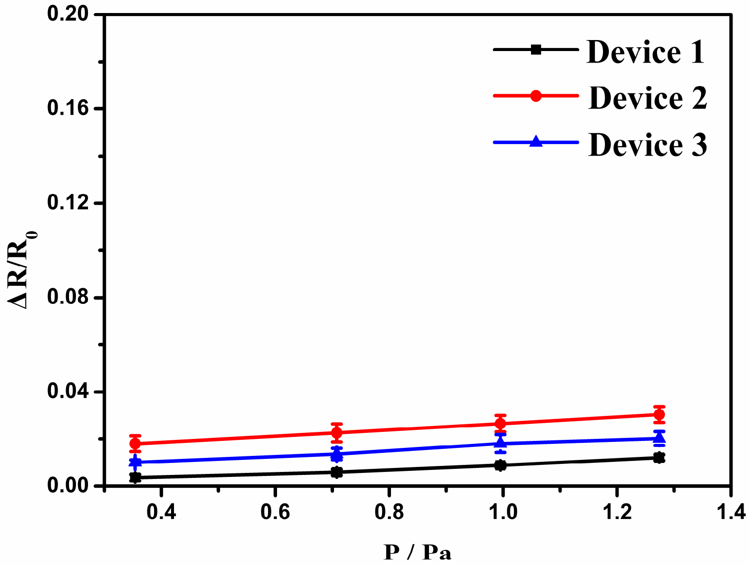

Figure S11. Run-to-run difference of the fabrication process.

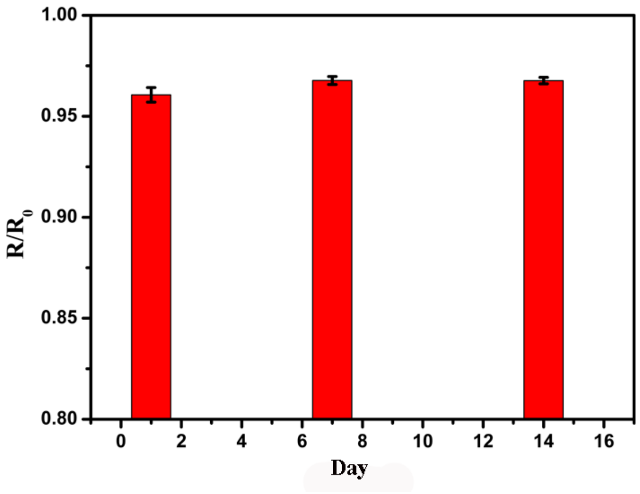

Figure S12. The long-term stability of the as-prepared device.

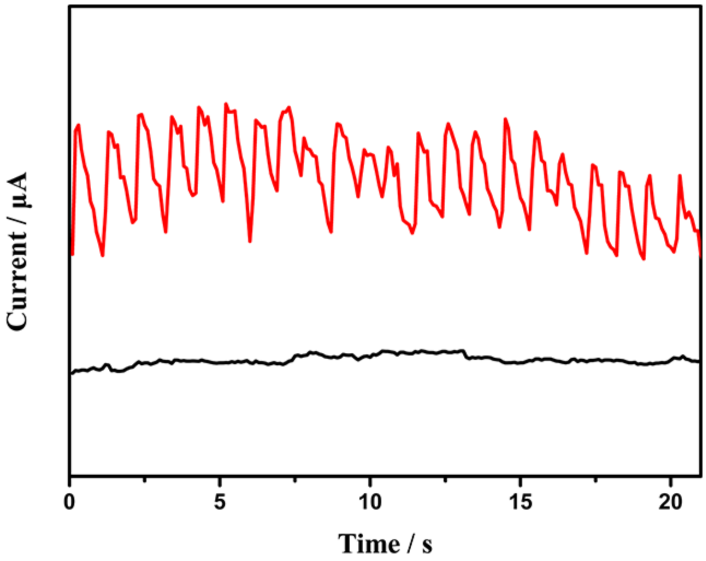

Figure S13. Real-time data of a device on wrist pulse region (red line) and other region (black line).
